# *Babesia divergens* IgG Antibodies and ABO Blood Types in Humans

**DOI:** 10.3390/pathogens15101016

**Published:** 2026-09-25

**Authors:** Ingrid Alm Hirsch, Joel Svensson, Muyideen Kolapo Tijani, Lena Danielsson, Jill R. Storry, Kristina E. M. Persson

**Affiliations:** 1 Division of Clinical Chemistry and Pharmacology, Department of Laboratory Medicine, Lund University, 22185 Lund, Swedenkristina.persson@med.lu.se (K.E.M.P.); 2 Clinical Chemistry and Pharmacology, Lund University Hospital, Region Skåne, 22185 Lund, Sweden; 3 Laboratory Medicine, Unilabs, Skaraborg Hospital Skövde, 54949 Skövde, Sweden; 4 Skaraborg institute for Research and Development, 54130 Skövde, Sweden; 5 Department of Clinical Immunology and Transfusion Medicine, Lund University Hospital, Region Skåne, 22185 Lund, Sweden; jill.storry@med.lu.se; 6 Division of Transfusion Medicine, Department of Laboratory Medicine, Lund University, 22185 Lund, Sweden

**Keywords:** *Babesia divergens*, ABO blood groups, blood group preference, ELISA

## Abstract

*Babesia* is an intraerythrocytic parasite transmitted by ticks or through blood transfusion, and it can cause babesiosis with symptoms such as fever, anemia, and organ failure. The parasite is closely related to the mosquito-borne *Plasmodium falciparum*, which causes malaria. Several studies have shown that individuals with blood group O have a reduced risk of severe malaria compared to individuals with other ABO blood groups. Whether or not this is also the case for babesiosis has not yet been clearly investigated. In this study, serum samples from over 650 individuals were analyzed using an ELISA assay to detect antibodies against *Babesia divergens*. The ABO blood groups of the same samples were determined using a conventional method. There was no difference in *B. divergens* IgG antibody levels between the different ABO blood groups, nor was there any difference in the percentage of positive reactions in the ELISA against *B. divergens* between the different blood groups. This could indicate that the development and maintenance of *B. divergens* antibodies are not influenced by ABO blood groups.

## 1. Introduction

Human babesiosis is a zoonotic disease caused by several malaria-like intraerythrocytic *Babesia* species. It is primarily transmitted through tick bites but can also be spread via blood transfusions; transplacental transmission has been reported in rare cases [1,2]. In Europe, *Babesia divergens* (*B. divergens)* is the most commonly reported species causing human disease, although *B. venatorum*, *B. crassa*-like organisms, and *B. microti* have also been identified [3,4]. In Sweden, *B. divergens*, *B. venatorum*, and *B. microti* have been detected in questing ticks [5]. Furthermore, among individuals in southern Sweden who were seropositive for the tick-borne pathogen *Borrelia burgdorferi*, the prevalence of antibodies against *Babesia* was reported to be as high as 16% [6]. This finding is consistent with the documented occurrence of *Babesia* spp. in both feeding and questing *Ixodes ricinus* ticks—the principal vector of tick-borne pathogens in Europe—collected from the same geographical region [7].

*B. divergens* infection is typically asymptomatic in immunocompetent individuals but may present with clinical manifestations such as fever, chills, and hemolytic anemia [8]. The risk of severe disease, including organ failure and death, increases with age, immunosuppression, and impaired splenic function [3,9].

ABO blood groups have been associated with differences in susceptibility and disease severity across several human infectious diseases, including SARS-CoV-2 infection, *Escherichia coli* infection, and peptic ulcer disease [10,11]. A notable example is malaria caused by the intraerythrocytic parasite *Plasmodium falciparum*, where individuals with blood group O have a substantially lower risk of severe disease than those with blood group A, B, or AB [12,13]. This protective effect is thought to have evolutionarily contributed to the higher prevalence of blood group O in malaria-endemic regions [14].

In the United States, *B. microti* is the most diagnosed *Babesia* species [15]. A clinical study conducted across 10 endemic northeastern states found that individuals expressing the RhD antigen may have a lower risk of *B. microti* infection and severe babesiosis. However, no clear association between ABO blood groups and *B. microti* infection was identified [16]. More recently, *B. divergens* was shown to grow equally well in vitro in erythrocytes from all ABO blood groups [17]. Nevertheless, *Ixodes ricinus*, the predominant tick species in Europe and Sweden and the principal vector of *Babesia* parasites, has been shown in a pilot study to be more attracted to individuals with blood group A [18]. Given that many *B. divergens* infections are asymptomatic or present with nonspecific symptoms and therefore may remain undetected, serological methods have become valuable tools for assessing previous exposure to *Babesia* spp. in diverse European populations [6,19,20,21]. Most seroprevalence studies have relied on indirect immunofluorescence assays (IFAs), which are labor-intensive and require considerable expertise for accurate interpretation. We previously developed an ELISA based on crude antigens prepared from purified *B. divergens* merozoites and demonstrated a strong correlation with the conventional IFA method [22].

Using the developed ELISA method, the aim of this study was to determine the prevalence and levels of antibodies against *B. divergens* among individuals with different ABO blood groups in Sweden, thereby assessing whether naturally acquired antibodies, which may indicate current or past infection, vary according to blood group.

## 2. Materials and Methods

### 2.1. Sample Collection and Areas

Serum samples from the Biological Specimen Bank of Clinical Microbiology, Skåne University Hospital, Lund, Sweden, collected during 2018–2019 and positive for antibodies against *Borrelia burgdorferi* sensu lato (*Borrelia*-positive), were obtained from 300 individuals. In addition, serum samples collected from 360 individuals who visited their general practitioners at healthcare centers (HCs) for various symptoms during the late summer months of 2017 were obtained anonymously (HC anonymous). The selection of HC anonymous samples was performed at the hospital laboratories in Kristianstad and Hässleholm, Southern Sweden, based on the site of sampling; neither age nor sex was considered. Serum samples were also collected from 112 healthy individuals, who served as a reference group. All serum samples were stored at −80 °C.

Ethical permissions for the above studies were obtained from the Regional Ethical Board (2017/177), Lund, Sweden, and from the Swedish Ethical Review Board (2020-06789).

### 2.2. Merozoite Extract Preparation

Crude merozoite antigen was prepared from the *Babesia divergens* Lund1 strain as previously described [22]. Briefly, culture supernatants from O+ erythrocytes with a parasitemia of 30–40% were collected following centrifugation at 1217× *g* for 3 min. The pooled supernatants were transferred to 50 mL Falcon tubes and centrifuged at 5445× *g* for 12 min to pellet the merozoites. The resulting pellet was resuspended in 5–10 mL of the remaining supernatant and passed through a 1.2 µm Acrodisc 32 mm syringe filter (Pall Corporation, Port Washington, NY, USA). The filtered merozoites were subsequently washed three to four times with PBS by centrifugation at 19,000× *g* for 7 min per wash. After the final wash, the merozoite pellet was resuspended in 100 µL PBS. A relatively homogeneous antigen extract was generated by subjecting the merozoites to four freeze–thaw cycles. The protein concentration of the resulting preparation was then measured at 280 nm using a NanoDrop spectrophotometer assuming A_280_, 0.1% of 1.0.

### 2.3. Anti-B. divergens IgG ELISA

The ELISA method used for the measurement of antibodies against *B. divergens* has been described previously [22]. Briefly, MaxiSorp Immunoplates (Thermo Fisher Scientific, Waltham, MA, USA)) were coated overnight at 4 °C with 50 µL/well of merozoite extract in PBS (1.1 µg protein/well). The plates were washed three times with 200 µL/well of wash buffer (PBS containing 0.05% Tween 20) and then blocked with 200 µL/well of 1% casein in PBS (Thermo Fisher Scientific, Waltham, MA, USA) for 2 h at room temperature (RT). After washing three times, 50 µL of serum sample diluted 1:50 in dilution buffer (0.1% casein in wash buffer) was added to each well. The plates were incubated for 2 h at RT and washed three times, and then 50 µL/well of rabbit anti-human IgG heavy-chain antibody (Thermo Fisher Scientific, Waltham, MA, USA) diluted 1:10,000 in dilution buffer was added and incubated for 1 h at RT, followed by washing three times. Next, 50 µL/well of goat anti-rabbit IgG (H + L)-HRP (Bio-Rad, Hercules, CA, USA), diluted 1:3000 in dilution buffer, was added and incubated for 1 h at RT. The plates were then washed three times, and 100 µL/well of TMB One Solution (Promega, Madison, WI, USA) was added and incubated for 4 min, after which the reaction was stopped by 100 µL/well of 1 M H_2_SO_4_. Absorbance was measured at 450 nm within 30 min using a Multiskan Sky microplate reader (Thermo Fisher Scientific, Waltham, MA, USA). Each plate included blank, positive, and negative controls to enable comparison between plates, and the mean absorbance value for each serum sample was calculated from duplicate measurements.

In order to exclude the possibility that a high absorbance value on the ELISA was due to a positive reaction for casein, the samples that were classified as positive in the ELISA were also tested on a similar ELISA, except that the plates were coated with 50 µL/well of 1% casein in PBS.

To investigate the possibility that a positive reaction in the ELISA was unspecific and due to rheumatoid factor (RF), 6 of the serum samples (4 of which showed very high absorbance values and 2 of which showed very low values in the ELISA) were tested for rheumatoid factor (RF). The RF test was conducted with an immunoassay detecting RF IgM antibodies with fluorometry in the hospital routine laboratory at Clinical Immunology and Transfusion medicine, Lund, Region Skåne, using a method calibrated to the WHO reference preparation W1066.

### 2.4. ABO Blood Type Testing

Plasma was examined for the presence of anti-A and/or anti-B: each sample was tested against A_1_ and B erythrocytes using NaCl enzyme test/cold agglutinin cards (Bio-Rad, Hercules, CA, USA, cat.# 005014) following the manufacturer’s instructions.

### 2.5. Statistical Analysis

Microsoft Excel, version 16.72 (Microsoft Corporation, Redmond, WA, USA), was used for data organization and curation. R (version 4.6.1) [23] was used for statistical analyses and graph production. The Kruskal–Wallis test was used to compare antibody levels between the ABO blood groups. Fisher’s exact test was used to compare the proportions of individuals seropositive for *B. divergens* antibodies between the ABO blood groups. Differences in other proportions were assessed using Pearson’s chi-squared test. For all comparisons of proportions, the effect size was quantified by Cramer’s V, where values of 0.1, 0.3, and 0.5 were considered small, moderate, and large effects, respectively. A statistical significance level of *p* = 0.05 was used in all statistical calculations.

## 3. Results

### 3.1. Demographic Data

Of the 300 *Borrelia*-positive individuals, 141 (47%) were male, and 159 (53%) were female (Table 1). The reference group consisted of 112 individuals, of whom 47 (42%) were male, and 65 (58%) were female (Table 1). Participants in the reference group ranged in age from 18 to 66 years. There was no information on sex or age for the HC anonymous cohort.

### 3.2. Distribution of Blood Groups Between Borrelia-Positive and HC Anonymous Cohorts

When the ABO blood group distribution in the cohorts was compared, it was found that there was no statistically significant difference in the distribution of ABO blood groups between the *Borrelia*-positive and HC anonymous cohorts (χ^2^(3) = 2.75, *p* = 0.43, Cramer’s V = 0.065). The ABO distribution in the cohorts also did not differ from that in the general Swedish population according to [24] (Table 2) (χ^2^(3) = 7.23, *p* = 0.06, Cramer’s V = 0.001).

### 3.3. Test of Unspecific Reactivity

The ELISA described above was used to test for antibodies against *B. divergens*. To exclude the possibility that antibodies were unspecifically reacting to casein, which was used as a blocker, all samples with absorbance values above 1.5 (*n* = 52) in the *B. divergens* ELISA were also tested with plates coated with casein. One sample that reacted strongly to both the *Babesia* extract and casein was excluded from further analyses. None of the six serum samples analyzed for rheumatoid factor (RF) gave positive results for RF.

### 3.4. Comparison of Level of Antibodies Against B. divergens in ABO Blood Groups

There were no significant differences in the median anti-*B. divergens* IgG levels between different blood groups (*p* = 0.80) (Figure 1). The medians in the different blood groups were distributed as follows (95% confidence interval): blood group A: 0.41 (0.37–0.45), blood group AB: 0.34 (0.22–0.52), blood group B: 0.40 (0.33–0.47), and blood group 0: 0.38 (0.32–0.45).

### 3.5. Comparison of Seroprevalence of B. divergens IgG Antibodies in the ABO Blood Groups

Serum samples from 112 healthy individuals were used as a reference group to establish the cut-off for the positivity of cohort samples for *B. divergens* IgG antibodies, based on the mean and standard deviations (SD) of the absorbances obtained from the reference group. See Supplementary Figure S1 for the distributions of the reference group and the cohorts. An absorbance value ≥1.77 (mean + 2 SD) but <2.35 (mean + 3 SD) was considered weakly positive. An absorbance value ≥2.35 (mean + 3 SD) was considered strongly positive.

In total, 37 of the 659 tested individuals (5.6%) had *B. divergens* antibodies (absorbance values ≥1.77). Of these, 18 individuals (2.7%) tested weakly positive, and 19 individuals (2.9%) tested strongly positive (Table 3). A comparison between the different blood groups using Fisher’s exact test revealed no statistically significant difference between the proportions of individuals testing positive for *B. divergens* antibodies (*p* = 0.43, Cramer’s V = 0.066). Also, no statistically significant difference was found when the comparison was based on the proportion of weakly positive only (*p* = 0.36, Cramer’s V = 0.071) or strongly positive only (*p* = 0.84, Cramer’s V = 0.027).

Due to possible differences in exposure to tick bites between the cohorts, *B. divergens* antibody levels and seroprevalence were compared within each cohort. There were no significant differences in *B. divergens* antibody levels among the ABO blood groups in either the *Borrelia*-positive cohort (*p* = 0.49) or the HC anonymous cohort (*p* = 0.11). Furthermore, when the baseline antibody level of the reference group was used to determine seropositivity, the proportions of individuals who were negative or positive for *B. divergens* did not differ significantly among the ABO blood groups in either the *Borrelia*-positive cohort (*p* = 0.053, Cramer’s V = 0.18) or the HC anonymous cohort (*p* = 1.0, Cramer’s V = 0.025).

## 4. Discussion

We investigated the acquisition of *Babesia divergens*-specific IgG antibodies in cohorts comprising individuals with *Borrelia*-positive serology and patients attending healthcare centers for various clinical conditions. We confirmed that the ABO blood group distribution of these cohorts closely mirrored that of the Swedish population [24], indicating that the blood group distribution of the study population is representative of that of the national population.

We detected no statistically significant association between ABO blood group and anti-B. *divergens* IgG levels. This contrasts with findings from Papua New Guinea, where children with blood group O exhibited higher antibody levels against *P. falciparum* variant surface antigens, predominantly PfEMP1 [25]. One possible explanation for this is the nature of the antigens examined. In our study, ELISA plates were coated with crude merozoite antigens, whereas different results might be obtained when using antibodies directed against Variant Erythrocyte Surface Antigen 1 (VESA1), a highly polymorphic functional ortholog of PfEMP1 found in *B. divergens* and other *Babesia* species, but not in *B. microti* [26,27].

When anti-*B. divergens* IgG levels were classified according to a reference-group cut-off, seroprevalence remained comparable among the ABO blood groups. This finding suggests that individuals with different blood groups are equally able to acquire *B. divergens* IgG antibodies. Although one study has reported that ticks preferentially feed on individuals with blood group A [18], this finding has not been confirmed in large-scale field studies, and there is currently insufficient evidence to conclude that ABO blood group substantially influences tick bite risk in humans. Meanwhile, a clinical study conducted in a babesiosis-endemic region of the United States found no association between ABO blood group and the risk of *B. microti* infection, high parasitemia, or severe disease requiring intensive care. Consistent with these observations, we previously demonstrated that a *B. divergens* isolate showed no preference for erythrocytes of any ABO blood group in vitro [17].

In malaria, blood group O erythrocytes are associated with reduced rosette formation and protection against severe disease [13]. This effect is attributed to weaker interactions between blood group O erythrocytes and RIFINs, a variant surface antigen expressed by *P. falciparum*-infected erythrocytes [28]. Although severe *B. microti* infection has not been associated with ABO blood group, similar clinical studies are needed for *B. divergens* babesiosis because of important genomic differences in their variant surface antigens, including VESA1 [26,27]. Such investigations are particularly relevant, as the factors that predispose certain individuals to develop severe symptoms from the generally asymptomatic *B. divergens* infection remain poorly understood.

Due to the high degree of antigenic conservation among closely related *Babesia* species, cross-reactive antibody responses cannot be excluded. We previously showed that the *B. divergens* merozoite antigen used in this assay reacted with serum from a patient infected with a crass-like *Babesia*. Therefore, seropositivity in the present study may not exclusively reflect exposure to *B. divergens* but could, in some cases, represent exposure to other closely related *Babesia* species, which is a limitation of the study.

This study has other limitations. First, it was difficult to determine whether the two cohorts had comparable levels of tick bite exposure, and the lack of demographic information for the HC anonymous cohort limited further analyses. Second, the absence of participants with documented *B. divergens* infection prevented assessment of the acquisition and persistence of antibodies across ABO blood groups. Finally, it was challenging to establish an ideal reference group consisting of individuals with well-documented histories of no prior exposure to *Babesia* parasites. As a result, the ELISA cut-off used to define seroreactivity may have been inflated by the inclusion of individuals with previous, unrecognized exposure. This could have contributed to the relatively small number of seropositive individuals identified in the study and reduced the statistical power to detect differences among ABO blood groups.

In conclusion, no association between ABO group and anti-*B. divergens* seroreactivity was detected in the studied populations. Since serological responses may reflect previous exposure and may be influenced by cross-reactivity, further studies are needed to confirm whether ABO blood group influences susceptibility to *B. divergens* infection.

## Figures and Tables

**Figure 1:**
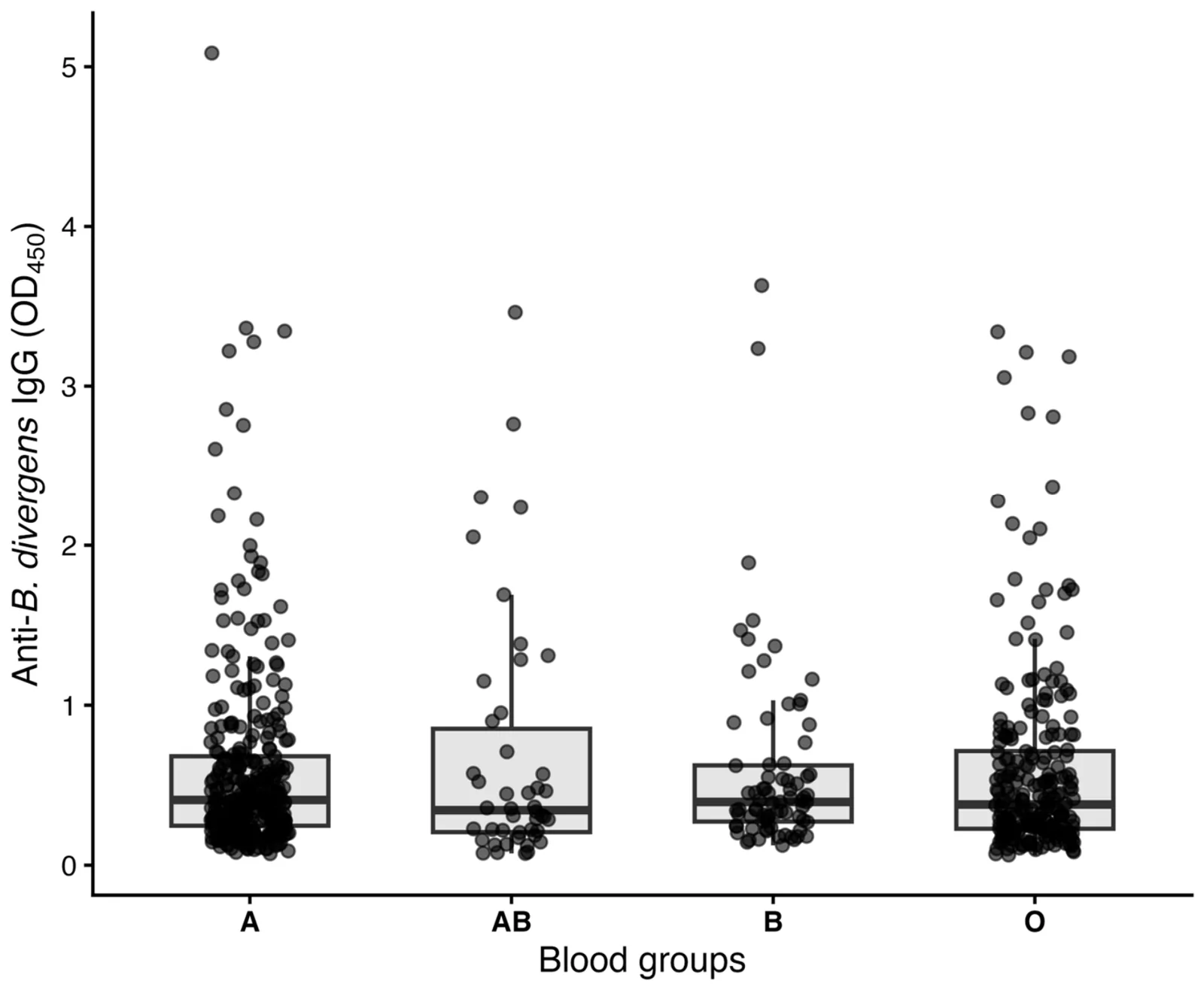
Comparison of median absorbances of antibodies produced against *B. divergens* between the different blood groups. The boxes show the lower quartile, the median, and the upper quartile values of the absorbance measured. The Kruskal–Wallis test yielded no statistically significant difference in antibody levels between the blood groups (*p* = 0.80).

**Table 1:** Description of the cohorts and the reference group. n/a, not available.

	*n*	Age (Years)	Male/Female (%)
*Borrelia*-positive	300	3–92	47/53
HC anonymous	359	n/a	n/a
Healthy reference group	112	18–66	42/58
Total	771		

**Table 2:** The distribution of blood groups among tested individuals, % (*n*).

	A	B	AB	O
*Borrelia*-positive (*n* = 300)	45 (136)	13 (39)	6 (18)	36 (107)
HC anonymous (*n* = 359)	48 (173)	10 (37)	8 (28)	34 (121)
All individuals (*n* = 659)	47 (309)	11 (76)	7 (46)	35 (228)
Swedish reference population (*n* = 4,204,234) [24]	47	10	5	38

**Table 3:** ELISA results for comparison of presence of antibodies against *B. divergens* between the ABO blood groups showed no differences between blood groups.

Blood Group	*n*	Positive (≥Mean + 2 SD), %	Weakly Positive (≥Mean + 2 SD but <Mean + 3 SD), %	Strongly Positive (≥Mean + 3 SD), %
A	309	5.5 (*n* = 17)	2.9 (*n* = 9)	2.6 (*n* = 8)
B	76	3.9 (*n* = 3)	1.3 (*n* = 1)	2.6 (*n* = 2)
AB	46	10.9 (*n* = 5)	6.5 (*n* = 3)	4.3 (*n* = 2)
O	228	5.3 (*n* = 12)	2.2 (*n* = 5)	3.1 (*n* = 7)
All	659	5.6 (*n* = 37)	2.7 (*n* = 18)	2.9 (*n* = 19)

## Data Availability

All data are available through the corresponding author.

## Supplementary Materials

The following supporting information can be downloaded at: https://www.mdpi.com/article/10.3390/pathogens15101016/s1, Figure S1: A violin plot (with jittered points) showing the distribution of the absorbances of the cohorts: *Borrelia*-positive (mean = 0.69, median = 0.54), reference (mean = 0.62, median = 0.42), and HC anonymous (mean = 0.54, median = 0.31). The mean is indicated by a red circle and the median by a blue polygon.
