# Supplementary material for: Babesia divergens IgG Antibodies and ABO Blood Types in Humans
Source: Pathogens. 2026 Sep 25;15(10):1016. doi: 10.3390/pathogens15101016 (PMC13708286; doi:10.3390/pathogens15101016)
Supplement: Supplementary file 1 [file pathogens-15-01016-s001.zip › pathogens-4532934-supplementary.pdf]

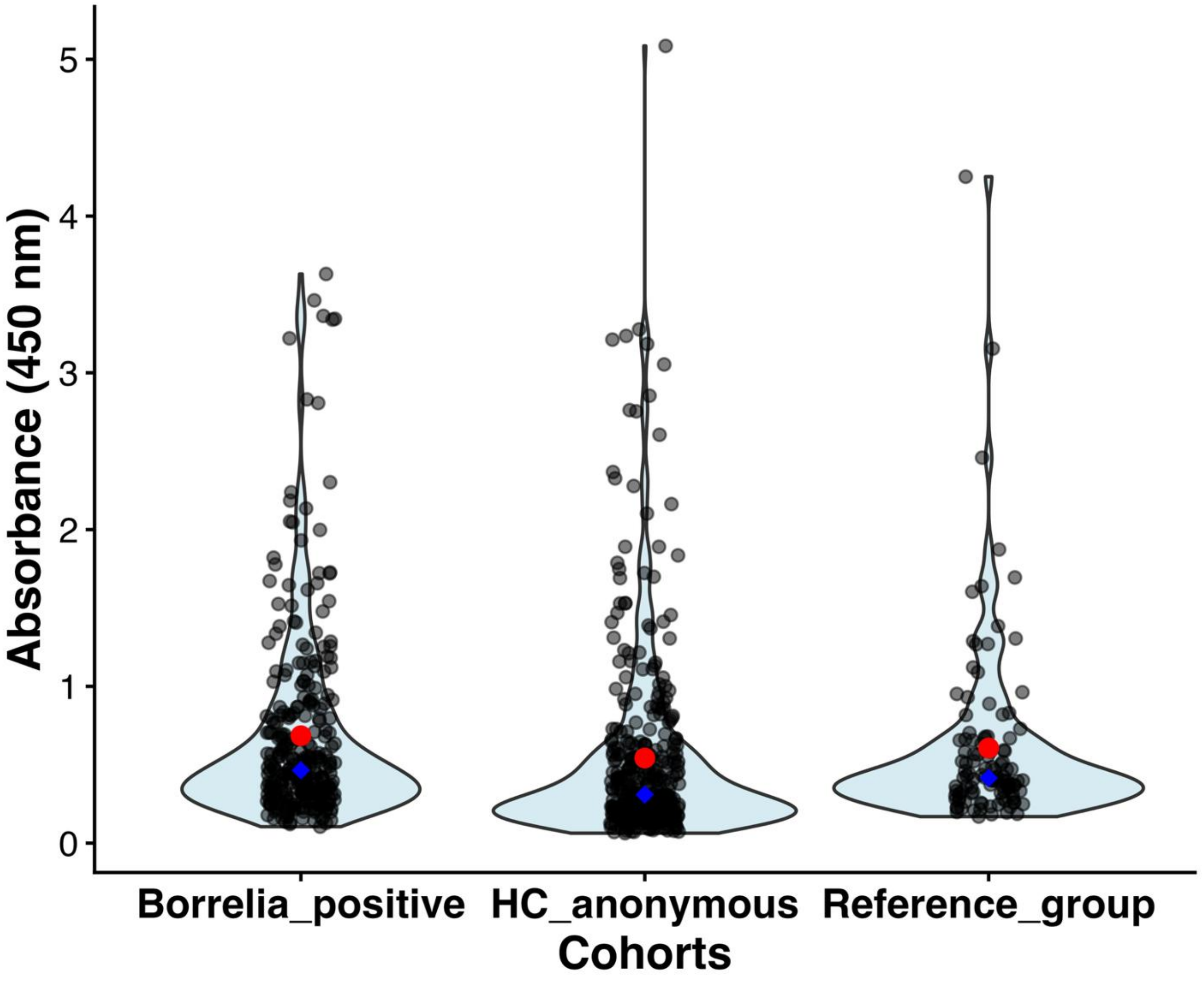

Supplementary figure s1: A violin plot (with jittered points) showing the distribution of the absorbances of the cohorts: *Borrelia*-positive (mean = 0.69 , median = 0.54), Reference (mean = 0.62, median = 0.42), and HC anonymous (mean = 0.54, median = 0.31). The mean is indicated by a red circle and the median by a blue polygon.
